# Tibial and femoral osteotomies in varus deformities - radiological and clinical outcome

**DOI:** 10.1186/s12891-020-03232-2

**Published:** 2020-03-31

**Authors:** Julian Fürmetz, Sven Patzler, Florian Wolf, Nikolaus Degen, Wolf Christian Prall, Chris Soo, Wolfgang Böcker, Peter Helmut Thaller

**Affiliations:** 1 3D-Surgery, Department of General, Trauma and Reconstructive Surgery, University of Munich LMU, Nußbaumstraße 20, 80336 München, Germany; 2 Department of General, Trauma and Reconstructive Surgery, University of Munich LMU, Munich, Germany; 3 Paracelsus Medical University PMU, Salzburg, Austria; 4 Faculty of Health Sciences and Medicine, Bond University, Gold Coast, QLD Australia; 5 School of Medicine, Griffith University, QLD, Gold Coast, Australia

**Keywords:** Osteotomies, Distal femoral osteotomy, DFO, High Tibial osteotomy, HTO, Valgisation, Realignment, Varus deformities, Medial osteoarthritis

## Abstract

**Background:**

Varus deformities of the knee are frequently corrected by osteotomies, which should be performed at the level of origin. But in contrast to high tibial osteotomies (HTO), little data exists for distal femoral osteotomies (DFO).

This study evaluates radiological and clinical outcomes after valgisation osteotomies in the proximal tibia and distal femur.

**Methods:**

We used an observational cohort study design and prospectively performed preoperative long standing radiographs (LSR), lateral x-rays and clinical questionnaires (SF-36, Lysholm score, VAS). Postoperative LSR and lateral x-rays were obtained on average 18 months postoperative and postoperative clinical questionnaires at final visit (mean follow up 46 months). A subgroup analysis of the different surgical techniques (oHTO vs. cDFO) was performed, with regards to radiological and clinical outcomes.

**Results:**

Finally 28 osteotomies with medial tibial opening (oHTO) or lateral femoral closing (cDFO) wedge osteotomies in 25 consecutive patients (mean age 40 years) were identified. There were 17 tibal and 11 femoral procedures. All osteotomies were performed at the origin of deformity, which was of different etiology. The average deviation of the final HKA compared to the preoperative planning was 2.4° ± 0.4°. Overall, there was a significant improvement in all clinical scores (SF-36: 61.8 to 79.4, *p* < 0.001; Lysholm-score: 72.7 to 90.4, *p* < 0.001; VAS: 3 to 1, *p* < 0.001). There was no significant correlation between surgical accuracy and outcome scores.

**Conclusion:**

Valgisation osteotomies lead to a significant improvement in all clinical scores with the demonstrated treatment protocol. An appreciable proportion of varus deformities are of femoral origin. Since cDFO provides comparable radiological and clinical results as oHTO, this is an important treatment option for varus deformities of femoral origin.

## Background

Varus malalignment has been identified as a risk factor for the incidence and progression of medial osteoarthritis (OA) [1]. Deformity correction with osteotomies near the knee joint is therefore an important therapeutic intervention, which may prevent or delay the need for joint replacement even in cases of severe cartilage damage independent of patient age [2]. This is especially relevant in younger patients, as lifetime risk of revision surgery after knee joint replacements increases dramatically within this patient group [3].

In varus deformities, osteotomies were usually performed in the tibia, with valgus deformities predominantly treated with femoral procedures. However, it has become common practice to perform a deformity analysis using a long standing radiograph (LSR) to determine the origin of deformity prior to surgery [4, 5], since varus deformities can be localized either in the tibia or in the femur [6–8]. In the case of femoral malalignment, a high tibial osteotomy (HTO) results in a pathological oblique knee joint line with increased shear forces and vice versa in the case of a tibial malalignment and femoral correction [9]. Clinical and biomechanical studies indicate that if the postoperative knee joint line is not physiologically aligned, this leads to a poor result [7, 10, 11].

In contrast to the HTO, very little clinical data exist on lateral distal femoral osteotomies (DFO) in cases of varus deformities. At the distal femur, a closed wedge procedure is recommended due to the frequent instability in femoral open wedge osteotomies [12]. There exist only 2 studies reporting on lateral distal closing wedge femoral osteotomies, covering a total of only 19 cases [6, 8].

For the first time, this study evaluates radiological and clinical outcomes in valgisating femoral and tibial osteotomies.

## Methods

### Patients

Patients with symptomatic varus deformity treated with deformity correction (oHTO or cDFO) close to the knee joint were included in the study. Excluded were patients requiring simultaneous multilevel osteotomies or those with incomplete follow up.

In total, from 2009 to 2016, there were 28 osteotomies on 25 consecutive patients with varus deformities. The etiology was heterogenous: 9 congenital, 14 growth-related and 5 post-traumatic deformities. The demographic characteristics of patients including the BMI are presented in Table 1. Institutional review board approval was obtained for the study (EC-Nr.: 16–008). All involved patients gave their informed consent statement prior to the study inclusion.

**Table 1:** Demographic characteristics of patients

	oHTO	cDFO
Number of osteotomies (n (%))	17 (61%)	11 (39%)
Mean age at surgery (years ±SD (min-max))	37 ± 3 (18–61)	45 ± 4 (23–64)
Sex (m:f)	9:8	9:2
Mean BMI (kg/m^2^) at surgery (±SD (min-max))	25 ± 1 (17–30)	29 ± 2 (24–46)
BMI < 18,5 kg/m^2^ (n)	1	0
BMI 18,5–24,9 kg/m^2^ (n)	9	3
BMI > 25–29,9 kg/m^2^ (n)	7	6
BMI > 30 kg/m^2^ (n (%))	0	2
Mean follow-up (months (±SD))	41 ± 6	55 ± 8

### Standardised radiological and clinical assessment

**Fig. 1:**
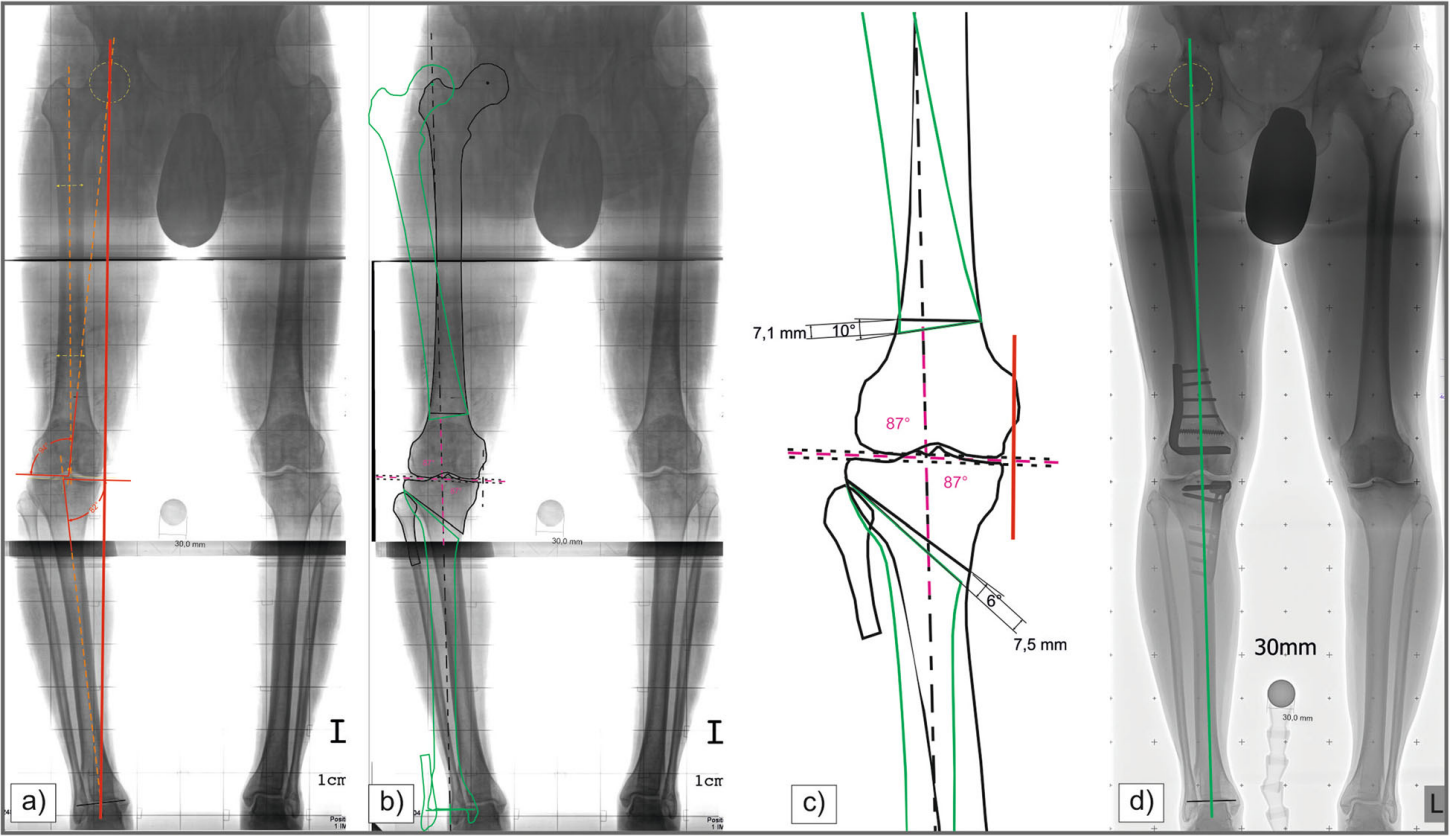
Two-staged femoral and tibial deformity correction in varus malalignment: **a** preoperative LSR with mechanical axis (red line), MPTA 82° and mLDFA 94°; **b** and **c** end point first planning with the new mechanical axis (black dotted line), 87° joint angles and preoperative mechanical axis (red short line); **d** final result after cDFO and oHTO with the new mechanical axis (green line)

Radiographic analysis of the pre- and postoperative LSR included the following parameters: mechanical axis deviation (MAD), hip knee angle (HKA), medial proximal tibial angle (MPTA), mechanical lateral distal femoral angle (mLDFA), joint line convergence angle (JLCA), patella height (Caton-Deschamps index), tibial slope, and posterior distal femoral angle (PDFA), according to the definitions by Paley [13]. For a better comparability to previous reports, we included the HKA. But in our daily clinical practice, joint angles, JLCA and MAD are the most important parameters for both our preoperative planning process and post-operative evaluation of the final result (Fig.1).

#### Preoperative

Preoperative planning was performed using the End Point First (EPF) planning method on long standing radiographs [14, 15]. Depending on the origin of deformity, the osteotomy site was chosen and lateral x-ray images of femur and/or tibia were added. In femoral malalignment (mLDFA > 90°), patients were treated with a lateral closed wedge distal femoral osteotomy (*n* = 11, 39%). In 17 knee joints (61%), analysis revealed a tibial deformity (MPTA < 85°), and medial open wedge high tibial osteotomy (oHTO) was performed.

Two patients underwent two-stage bifocal osteotomies. If possible, joint angles did not exceed normal values in preoperative planning (MPTA ≤90°, mLDFA ≥85°). However, in order to avoid a second intervention, some patients required a planned overcorrection, which were intended not to exceed 93 or 82 degrees respectively. To assess for functional and clinical status prior to admission, we used the Lysholm score, the, Short-Form-36 Health Survey (SF-36), and the Visual Analog Scale (VAS).

#### EPF - method

The EPF method starts with a malalignment test of the lower limb [4, 13]. The centers of the hip and ankle are determined, and a line is then drawn from the center of the hip joint to the center of the ankle joint. Next, tibial and femoral knee joint lines are drawn and the lateral distal femoral angle and medial proximal tibial angle are measured. In tibial deformities the new mechanical axis starts from the hip center and in femoral deformities from the ankle center and runs between the intercondylar tubercles. The aiming point of the new mechanical axis is between the medial and lateral intercondylar tubercle depending on cartilage and meniscal tears. After the osteotomy is located on paper or on a digital platform, the proximal part of the femur/distal part of the tibia is moved to the final location of the femoral head/ankle center located on the mechanical axis. In bifocal deformities a vertical line is drawn such that it forms an 87-degree lateral angle with the distal femoral joint line. This will subsequently be the new mechanical axis of the entire leg (Fig. 1).

#### Intraoperative

The same surgeon operated on all the patients. Intraoperative alignment control was performed with the x-ray grid, a 3 mm thin phenolic resin hard paper plate with intersected distinguishable radiopaque reference lines for determination of the mechanical axis. At the beginning of the procedure, meniscal and cartilage lesions were evaluated with arthroscopy. There were 4 meniscal partial resections and no cartilage intervention. Only TomoFix (Synthes, Oberdorf, Switzerland) plates were used as implants for the oHTO and the operative technique was similar to Staubli et al. with biplanar cutting technique [16]. We used a 95° blade plate (Synthes, Oberdorf, Switzerland) for femoral fixation. Operative technique for cDFO was similar to Lobenhoffer et al. [12].

#### Postoperative

Postoperative standard treatment was partial weight bearing (20 kg) for 6 weeks and regular physiotherapy.

On average, final radiological examination took place 6 months after implant removal, including LSR and lateral x-ray, which was generally 18 months postoperative. Mean follow up for clinical examination including questionnaires (Lysholm score, SF-36, VAS) was 47 months postoperatively (Tab. 2), with a minimum of 24 months.

**Table 2:** Clinical results of corrections within and beyond the normal range of 85–90° MPTA / mLDFA

			SF-36		Lysholm-Score	
		n (%)	preoperative	postoperative	preoperative	postoperative
**oHTO**	normal (MPTA < 90°)	10 (56%)	63	79	76	94
	overcorrection (MPTA > 90°)	7 (44%)	57	77	69	84
**cDFO**	normal (mLDFA> 85°)	8 (73%)	64	83	76	97
	overcorrection (mLDFA < 85°)	3 (17%)	57	74	59	71

### Statistics

As test of significance, a two-sided Wilcoxon test for dependent groups was performed (SPSS version 25, SPSS Inc., Chicago/Illinois, USA) to evaluate changes in radiological and clinical parameters before and after surgery. Subgroup differences (oHTO vs. cDFO) were calculated with the Mann-Whitney U test.

## Results

### Radiological results

The average deviation of the final HKA compared to the preoperative planning was 2.4° ± 0.4°.

**Fig. 2:**
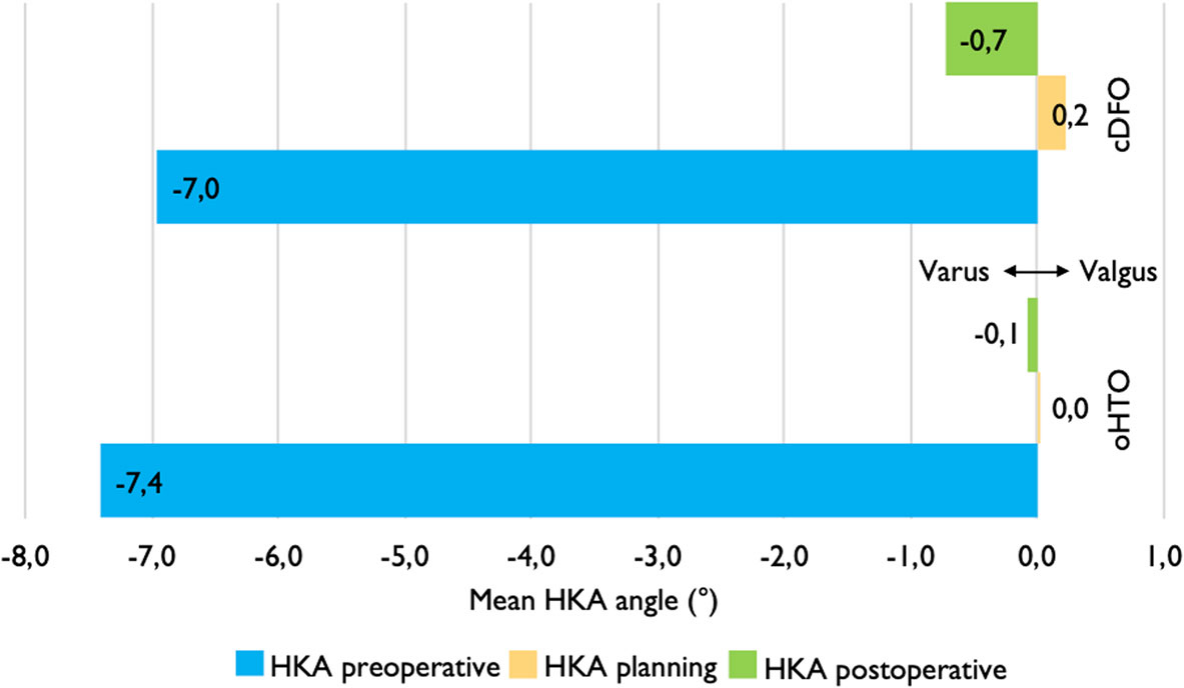
Mean preoperative HKA°(blue) of both subgroups, preoperative planning of HKA° (yellow) and final HKA° (green)

The preoperative HKA was on average − 7.4° ± 0.8 in the oHTO group and − 7.0° (SD ± 1.1) in the cDFO group, while the average amount of final correction was slightly higher in the oHTO group (7.3 ± 0.9°) versus the cDFO group (6.2 ± 1.6°) (final average HKA was − 0.1 vs. -0.7) (Fig. 2). The oHTO group had a slightly more precise correction result, with an absolute mean deviation of 2.2° ± 0.5 from preoperative planning, compared to the cDFO group with 2.6° SD ± 0.7. Accordingly, a deviation of less than ±3° was observed more frequently in the oHTO group after surgery (14 cases / 82%) than in the cDFO group (7 cases, 64%).

MAD changed from − 26.8 to − 0.1 mm on average in the oHTO group (*p* < 0.001), and slightly less in the cDFO group with − 24.4 to − 3.6 mm (*p* = 0.004). Differences between groups were not significant. Maximum preoperative MAD was − 44.4 mm in the oHTO group and − 49.8 mm in the cDFO group.

In tibial procedures, mean MPTA changed from 83.4° ± 0.7 to 90.2° ± 0.9 postoperatively (*p* < 0.001). Average preoperative planning of MPTA was 90.5° ± 0.6° (min. 85.6° - max. 93.0°), and the final absolute deviation from preoperative planning was 1.7 ± 0.4° (min. 0.2° - max. 5.9°). In 6 of 7 patients with MPTA (> 90°), planning of minor overcorrection was intended to address severe medial cartilage damage and to avoid bifocal osteotomies.

Due to the cDFO, the preoperative mLDFA decreased from an average of 92.1° ± 1.0 to 86.0° ± 0.7 (*p* = 0.003). The preoperative planning of mLDFA was 85.5 ± 0.6° (min. 83.4° - max. 89.5°) and the final absolute deviation from planning was 2.3 ± 0.4° (min. 0.3° - max. 4.6°). We identified 3 patients with overcorrected mLDFA (< 85°), of which all had severe medial cartilage damage and overcorrection was planned.

In the oHTO group, the JLCA decreased in average from − 2.1° ± 0.4 to − 1.6° ± 0.3 and in the cDFO group from − 2.3° ± 0.5 to − 1.9° ± 0.4. In both groups, leg length changed by 0.6 mm per degree of correction in MPTA / mLDFA. The oHTO increased tibial slope by an average of 0.8° ± 0.8 from 8.9° to 9.7° postoperatively. In contrast, the cDFO influenced the PDFA more clearly (by 4.9° ± 2.6) with an average preoperative PDFA of 83.8° to 88.8° postoperatively. A significant height reduction of the patella was observed only in the oHTO group (Caton Deschamps from 0.84 ± 0.02 to 0.74 ± 0.02; *p* < 0.05). No relevant changes were observed in the cDFO group (Caton Deschamps index from 0.79 ± 0.05 to 0.80 ± 0.08).

### Clinical results

**Fig. 3:**
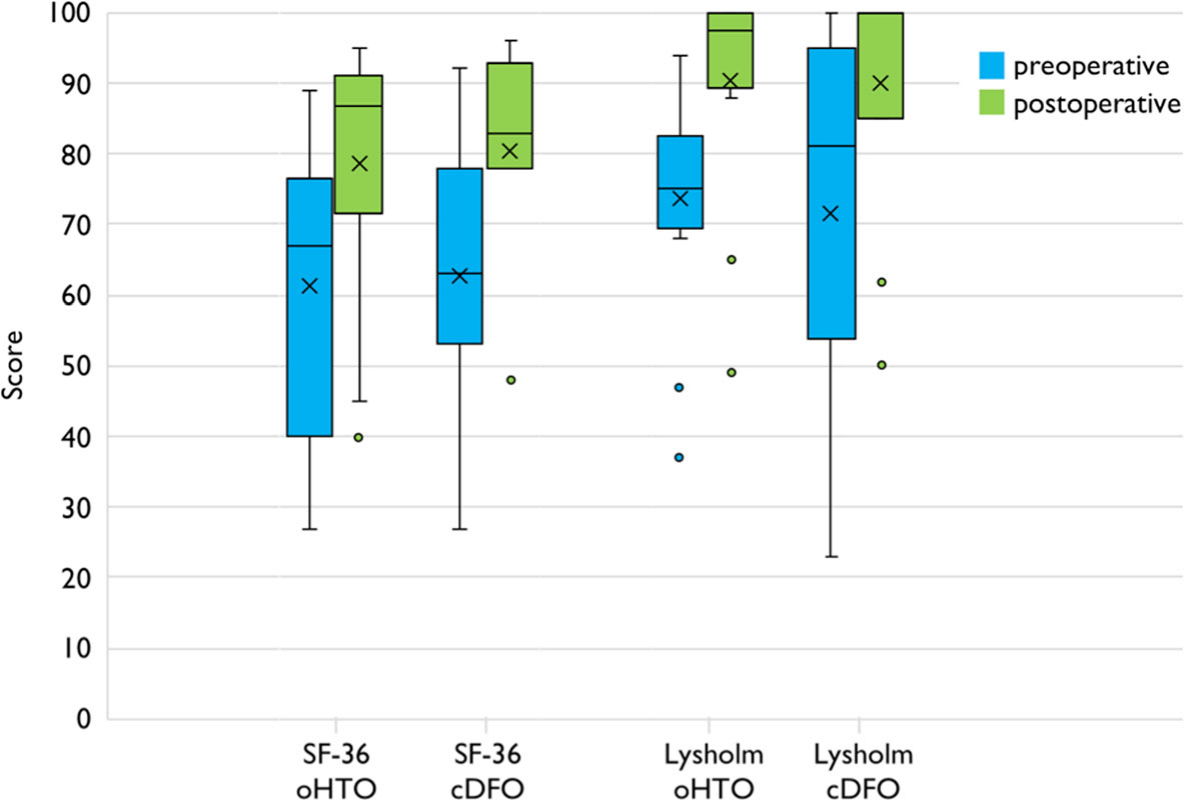
Boxplot of pre- and postoperative results of the SF- 36 and Lysholm-Score of both subgroups; x indicating the mean value

All clinical scores showed significant improvement at the final examination (mean follow up 47 months). The SF-36 quality of life score showed almost identical results for both groups, with the oHTO group achieving a significant improvement from 61.3 to 78.7 points (*p* < 0.001), and the cDFO group increasing from 62.6 to 80.5 (*p* = 0.005) (Fig. 3).

The Lysholm Score improved significantly in the oHTO group by an average of 16.9 points from 73.6 to 90.5 (*p* = 0.001). The cDFO group also achieved a similar result, with an average improvement of 19.6 points from 71.6 to 90.2 (*p* = 0.005) (Fig. 3).

The VAS improved in the cDFO group with an average of 2.5 points (SD ± 0.7; *p* = 0.003) and in the oHTO group with 1.9 points (SD ± 0.5 points; *p* = 0.001). In total, 64% of all patients reported complete absence of pain at the final examination.

Table 2 describes the results of the clinical scores for both groups pre- and postoperatively, distinguishing between overcorrections in MPTA/mLDFA compared to corrections within the normal range. It can be seen that the overcorrections have lower preoperative initial values and reach lower postoperative values, most likely reflecting a more severe cartilage damage in these patients.

### Clinical course and complications

There were no relevant differences in hospital stay, blood loss or surgery time. One occurrence of delayed bone formation in the oHTO group was successfully treated with autologous bone grafting.

## Discussion

This study evaluates radiological and clinical midterm outcome of re-alignment procedures in case of varus deformtities. The importance of comparing preoperative planning with actual postoperative alignment and the associated clinical outcome is emphasised. This allows to calculate the accuracy of the procedure with a mean deviation of 2.2° in the oHTO and 2.6° in dhe cDFO group in this study cohort.

### Mechanical axis and joint angles

The ideal degree of correction in cases of varus malalignment has been intensively discussed over many years. Several authors have identified a postoperative HKA of 3–5° valgus or a mechanical axis at 62–66% of the tibial width as optimal in medial OA [17–20]. Recently studies consider the extent of medial chondromalacia and perform an individually adjusted correction with a more moderate targeted range of valgus (HKA 1.7–5° or 50–65% of the total tibial plateau width), while avoiding overcorrection [4, 21]. A knee joint arthroscopy is recommended in the same session, for addressing intraoperative pathologies and fine-tuning of correction, depending on the type and extent of intraarticular damage [22]. Our approach corresponds to this and the average HKA and MAD of our patients indicate a postoperatively centered, and not a new, lateralised, mechanical axis. This is explained by the different etiologies in our study group, including younger patients without structural damage but with medial knee pain. For these patients the aiming point of the new mechanical axis is the medial intercondylar tubercle and for patient with grade IV medial cartilage degeneration (outerbridge classification) the lateral one.

Besides the new mechanical axis, joint angles are most important in preoperative planning. Overcorrection of the joint angles results in an oblique knee joint line with increased shear forces and poorer clinical outcome [7, 10].

But an overcorrection of MPTA (> 90°) or mLDFA (< 85°) was intended and performed in some of our patients with relevant OA. These patients had lower preoperative and postoperative values in all clinical scores. But still, the improvement of the scores was similar (Tab. 2). Therefore, it cannot be concluded from our data that overcorrections must be avoided and double level osteotomies should be performed, but other clinical and experimental data indicate this very strongly [7, 9, 10, 23, 24]. The maximum overcorrection that can be tolerated in femoral and tibial osteotomies should be examined more closely in further studies.

### Accuracy

Several HTO studies agree with a ± 3° deviation from planning as an acceptable range [25–28]. Reported results are very variable with 23 to 92% being in the defined target range [25–28]. 82% of our HTO-patients were within this range, so accuracy can be rated as good, but leaving room for improvement. To our knowledge, no results are available regarding accuracy of cDFO in literature. In our study, 64% (7 of 11) of the patients were within the ±3° limit of deviation with regards to preoperative planning. This is explained by the technically demanding closed wedge osteotomy, since the surgeon must rely on the accuracy of the bone resection, and intraoperative readjustment is only possible to a limited extent [6, 12]. Lateral inaccuracy of DFO could be produced by the same reason and due to the tension of the gastrocnemius muscle on the distal femur. The tibial slope influences the coronal alignment in long standing radiographs [29]. Sagittal changes in the distal femoral group may have influenced the coronal alignment and could count for some degree of inaccuracy.

There are various approaches to improve the accuracy of osteotomies. Simple and helpful in HTO is careful gap measurement [28]. Very promising results have recently been published by a single research group using patient-specific cutting guides in oHTO and oDFO [30, 31]. Another research group published improved results in medial cDFO for varization with 3D-printed patient-specific cutting guides [32]. This technique appears to be a promising option for both closing and opening wedge osteotomies to improve accuracy in the future, but there are still unsolved issues such as the complex and costly preoperative planning and printing process or the need for extensive bone exposure.

### Clinical outcome

Several authors report an improvement of clinical scores for up to 5 years postoperatively after HTO. The average postoperative Lysholm score is reported to range between 69 and 96 points and the mean SF-36 between 73 and 89 points [33–38]. Referring to those reports, the clinical results of our oHTO patients are within the upper range. Only the study by van der Woude et al. investigated the postoperative clinical outcome after a cDFO so far and reported a Lysholm score of 73 points and a pain level of 3 (VAS) [6]. In comparison, the patients in our cDFO group showed a 17-point higher Lysholm score and a 2-point lower postoperative pain level. Survival rates of the different treatment options diverge noticeably after a follow-up of 10 years. 5-year survival for oHTO was 90–99%, and was 94% for cDFO [6, 39]. After 10 years, the oHTO survival rate decreases to 64–92% [40]. A follow-up of more than 5 years for the cDFO is currently not described in literature.

### Limitations

Limitations of this study are the heterogeneous study population and the low case number for femoral and tibial osteotomies. The expected number of cases within this cohort and the mean values and standard deviations in accuracy and clinical outcome parameters in previous studies were too small for a prospective power analysis. Additionally, long-term information about clinical function or survival rates is missing.

High-volume studies from national or international databases with a focus on accuracy and resulting clinical function are necessary, because previous studies suggest that single clinical centers do not have sufficiently high case numbers to answer these questions. In our study group, over one third of the patients presented with varus malalignment of the distal femur and therefore were treated by distal femoral osteotomy. Reviewing the literature, distal femoral osteotomies are rarely described, which may reflect a high number of HTOs applied in cases with deformity at the distal femur. Similarly, in valgus deformities, the dogma that valgus = femur could already be disproved [40]. This reinforces our conviction that radiological evaluation (preoperative situation, planning and final result) must always be taken into account when evaluating the clinical results of osteotomies.

## Conclusion

Our results indicate that an appreciable proportion of varus deformities are of femoral origin and that cDFO provides comparable radiological and clinical results as oHTO. Through appropriate indication and patient selection, both kinds of valgisation osteotomies close to the knee joint can provide improvements in clinical function, pain level and quality of life. These joint-preserving interventions thus represent a valuable treatment option in varus deformities.

## Data Availability

The anonymised results of the radiological measurements and the clinical questionnaires are attached in the form of an Excel spreadsheet.

## Abbreviations

OA: Osteoarthritis
LSR: Long Standing Radiograph
HTO: High Tibial Osteotomy
DFO: Distal Femoral Osteotomy
BMI: Body Mass Index
MPTA: Medial proximal tibial angle
mLDFA: Mechanical lateral distal femoral angle
HKA: Hip knee angle
SF-36: Visual Analog Scale, Short-Form-36 Health Survey
MAD: Mechanical axis deviation
PDFA: Joint Line Convergence Angle, posterior distal femoral angle
